# Seasonal differences in baseline innate immune function are better explained by environment than annual cycle stage in a year‐round breeding tropical songbird

**DOI:** 10.1111/1365-2656.12948

**Published:** 2019-02-06

**Authors:** Chima J. Nwaogu, Will Cresswell, Maaike A. Versteegh, B. Irene Tieleman

**Affiliations:** ^1^ Groningen Institute for Evolutionary Life Sciences University of Groningen Groningen The Netherlands; ^2^ School of Biology University of St Andrews St Andrews Fife UK; ^3^ A.P. Leventis Ornithological Research Institute Jos Nigeria

**Keywords:** animal physiology, bird, ecological immunology, environmental change, individual variability, rainfall, seasonality and wild animals

## Abstract

1. Seasonal variation in innate immunity is often attributed to either temporal environmental variation or to life‐history trade‐offs that arise from specific annual cycle stages but decoupling them is difficult in natural populations.
2. Here, we effectively decouple seasonal environmental variation from annual cycle stage effects by exploiting cross‐seasonal breeding and moult in the tropical Common Bulbul *Pycnonotus barbatus*. We test how annual cycle stage interacts with a key seasonal environmental variable, rainfall, to determine immunity at population and individual level. If immune challenge varies with precipitation, we might expect immune function to be higher in the wet season due to increased environmental productivity. If breeding or moult imposes resource constraints on birds, depending on or independent of precipitation, we might expect lower immune indices during breeding or moult.
3. We sampled blood from 818 birds in four annual cycle stage categories: breeding, moult, simultaneous breeding and moulting, or neither. We quantified indices of innate immunity (haptoglobin, nitric oxide (NO_x_) and ovotransferrin concentrations, and haemagglutination and haemolysis titres) over two annual cycles of wet and dry seasons.
4. Environment (but not annual cycle stage or interactions between both) explained variation in all immune indices, except NO_x_. NO_x_ concentration differed between annual cycle stages but not between seasons. However, within the wet season, haptoglobin, NO_x_, ovotransferrin and haemolysis differed significantly between breeding and non‐breeding females. Aside from some recorded inconsistencies, population level results were largely similar to results within individuals that were measured repeatedly. Unexpectedly, most immune indices were higher in the dry season and during breeding.
5. Higher immune indices may be explained if fewer or poorer quality resources force birds to increase social contact, thereby exposing individuals to novel antigens and increased infection risk, independently of environmental productivity. Breeding birds may also show higher immunity if less immune‐competent and/or infected females omit breeding. We conclude that seasonal environmental variation impacts immunity more directly in natural animal populations than via resource trade‐offs. In addition, immune indices were more often variable within than among individuals, but some indices are characteristic of individuals, and so may offer selective advantages if heritable.

## Graphical abstract

Tropical environments are considered highly immune‐challenging for animals because of their relative higher organic productivity. However, this study from a seasonally arid tropical environment where annual cycle stages are decoupled from seasonal environmental variation, provides evidence that variation in innate immune function does not follow differences in annual cycle stages or simple environmental productivity pattern but may arise from interactions between individuals and their environment, and this may apply to variation in disease risk.

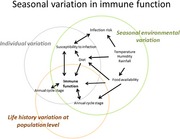

## 1 INTRODUCTION

Immune function varies seasonally (Dopico et al., 2015; Nelson & Demas, 1996), and this is often attributed to either variation in environment (Horrocks, Matson, & Tieleman, 2011; Tieleman, 2018) or to life‐history trade‐offs that are associated with resource allocation to competing annual cycle stages (Knowles, Nakagawa, & Sheldon, 2009; Sheldon & Verhulst, 1996). However, because annual cycle stages often covary with environmental factors, seasonal differences in immune function may reflect response to seasonal environmental variation, annual cycle stage or both. The difficulty in decoupling environmental effects (food, diet, vectors or antigenic properties) from life‐history‐related effects (resource cost of annual cycle stages or associated effects) sets limitations to interpreting immune variation on the basis of life‐history trade‐offs in natural populations (Pedersen & Babayan, 2011; Ricklefs & Wikelski, 2002; Schmid‐Hempel, 2003).

Interpreting immune variation on the basis of life‐history variation at the population level only (Hegemann, Matson, Both, & Tieleman, 2012; Tieleman, Williams, Ricklefs, & Klasing, 2005) may be problematic if individuals vary consistently in response to environment and/or life‐history challenges (Ardia, 2005b) due to differences in personality or quality (Araya‐Ajoy et al., 2018). Individuals may vary in exposure or tolerance to infection (Bansal, Grenfell, & Meyers, 2007; Lloyd‐Smith, Schreiber, Kopp, & Getz, 2005; Loehle, 1995) and in resource acquisition and allocation to immunity and other competing traits (van Noordwijk & de Jong, 1986; van de Pol & Wright, 2009). These differences may cause relationships between immune function and competing life‐history traits within individuals to be obscured at the population level by among‐individual variation (Merrill et al., 2015; Williams, Christians, Aiken, & Evanson, 1999). Therefore, understanding variation in immunity on the basis of life‐history trade‐offs requires an ecological context (Tieleman, 2018) where variation in both environment and individual animals is considered using carefully selected immune indices (Norris & Evans, 2000).

Seasonally arid tropical environments where resident birds breed and moult across different seasons are suitable for decoupling the effect of seasonal environmental variation from that of annual cycle stages on immune function. In Nigeria, there is a single wet and dry season annually, and environmental factors differ between and within these seasons. Food is usually more abundant in the wet season (Ngozi Molokwu, Olsson, Nilsson, & Ottosson, 2008; Nwaogu, Dietz, Tieleman, & Cresswell, 2017), but pathogens and disease vectors should also be more abundant in the wet season due to high environmental productivity (Pascual, Bouma, & Dobson, 2002; Young, Garvin, & McDonald, 1993). However, social contact may increase with less food and water during the dry season because animals cluster around fewer resources, and this may facilitate disease transmission (VanderWaal, Gilbertson, Okanga, Allan, & Craft, 2017), even if pathogen abundance is low. Furthermore, the effect of rainfall on environmental productivity may vary between seasons. For example, while the onset of the wet season is rapid, and the effects of precipitation are quickly felt, the rains do not end as abruptly, and hence, the commencement of the dry season, and associated drying out of the environment, is much more prolonged. Thus, conditions within the wet season should be less variable than the dry season. Nonetheless, the resident Common Bulbul *Pycnonotus barbatus* is capable of breeding year‐round (Cox et al., 2013; Nwaogu, Tieleman, & Cresswell, 2018) and its individuals are long‐lived (Stevens, Ottosson, McGregor, Brandt, & Cresswell, 2013) in central Nigeria. These characteristics make them ideal for decoupling the effects of environment and annual cycle stage on immune function. Some individuals moult into the dry season or overlap moult with breeding (Nwaogu et al., 2018), and this allows the effect of moult on immune function to be considered between seasons and breeding states. Residency allows repeat sampling of individuals between seasons and annual cycle stages. Consequently, temporal elevation of resource demands due to the occurrence of breeding and moult (Murphy, 1996; Sanz, Moreno, Merino, & Tomás, 2004) becomes effectively decoupled from seasonal environmental variation, allowing life‐history and environment effects to be decoupled at both population and individual levels.

Nonetheless, despite the large body of literature in ecological immunology, interpreting variation in immune indices is still ambiguous (Buehler, Versteegh, Matson, & Tieleman, 2011; Matson, Cohen, Klasing, Ricklefs, & Scheuerlein, 2006; Versteegh, Schwabl, Jaquier, & Tieleman, 2012). Depending on the immune index (Boughton, Joop, & Armitage, 2011; Demas, Zysling, Beechler, Muehlenbein, & French, 2011), high values may indicate a well‐protected animal capable of destroying invading agents, or a prior poorly protected and now highly challenged animal. From an “operative protection” point of view, high levels of immune indices should indicate increased investment proportionate to current or perceived infection (Horrocks, Matson et al., 2011), or trade‐offs due to increased investment in competing processes within the immune system (Martin, Weil, Kuhlman, & Nelson, 2006; McDade, Georgiev, & Kuzawa, 2016). Indices such as haemolytic or bacteria killing capacity of blood or plasma reflect ability to destroy foreign cells (Matson, Ricklefs, & Klasing, 2005; Tieleman et al., 2005) and thus a relative measure of an individual's ability to clear infection. But biomarkers of inflammatory response may have relative interpretations: haptoglobin is a positive acute phase protein which normally circulates in low concentration but increases with inflammation (Jain, Gautam, & Naseem, 2011; Matson, Horrocks, Versteegh, & Tieleman, 2012; van de Crommenacker et al., 2010; but see Hegemann, Matson, Versteegh, Villegas, & Tieleman, 2013). Ovotransferrin on the other hand is a negative acute phase protein. Both ovotransferrin and haptoglobin increase with inflammation because they bind to and remove haem from circulation during infection, so that haem is unavailable as nutrient to pathogens (Horrocks, Irene Tieleman, & Matson, 2011). But concentrations of ovotransferrin may decrease with increased inflammation because temporarily high free hormones may bind to ovotransferrin, and other acute phase proteins may be produced at the expense of ovotransferrin by the liver (Giansanti, Leboffe, Pitari, Ippoliti, & Antonini, 2012; Gruys, Toussaint, Niewold, & Koopmans, 2005; Jain et al., 2011). NOx modulates inflammatory processes but also participates in the direct killing of parasites and tumour cells (Sild & Hõrak, 2009). Overall, combining measures of haemolytic and natural antibody activity with multiple biomarkers of inflammatory response should give a robust assessment of constitutive immune function (Adamo, 2004).

In this study, we test the main and interactive explanatory power of seasonal environmental variation (i.e. occurrence of rainfall) and annual cycle stage on seasonal immune variation. We separate male and female bulbuls into annual cycle stages on the basis of breeding and moult occurrence and test differences in baseline innate immunity between the wet and dry season and within the wet season at population and individual levels. Specifically, we test: (a) the effects of seasonal environmental variation and annual cycle stage on variation in immune function of females and males at the population level; (b) the main and interactive effects of breeding and moult on immune function of females in the wet season (see justification for excluding males and dry season records in method section (below)); and (c) within individuals, the effect of seasonal environmental variation on immune function of non‐breeding and non‐moulting birds, and the separate effects of breeding and moult on immune function within the wet season. We expect immune indices to be higher in the wet season compared to the dry season due to higher environmental productivity, but we expect the occurrence of breeding and/or moult to lower immune function. However, differences between the wet and the dry season should remain consistent for different annual cycle stages in both sexes, if seasonal environmental variation is a more crucial determinant of seasonal variation in immune function. Otherwise, differences between annual cycle stages should be consistent between the wet and dry season at both population and individual levels. But, if both factors are important, we might expect interactions between them. Under such interaction scenarios, we predict that: (a) immune indices should be lowest for breeding or moulting birds in the dry season and highest for non‐breeding or non‐moulting birds in the wet season; (b) Breeding and moulting females should have lower immune indices compared to non‐breeders and non‐moulters, especially when breeding and moult overlap; and (c) Patterns within individuals should be similar to patterns at the population level and repeatability should be low, if the effect of seasonal environmental variation and/or annual cycle stage leads to larger variations within than among individuals.

## 2 MATERIALS AND METHODS

### 2.1 Birds, blood sampling and determination of variables

Field work was carried out at the Amurum Forest Reserve (09°52′N, 08°58′E) which is located at the A.P. Leventis Ornithological Research Institute (APLORI) on the Jos Plateau in north central Nigeria (see Supporting Information for more details on field site). All field work was approved by the APLORI scientific committee. We collected 818 blood samples from 530 individuals over 256 days between 29 January 2014 and 5 February 2016 (mean = 3.2, *SD* = 2.8, min = 1, max = 16 bulbuls/day, Supporting Information Figure S2). All birds were caught using mist nets between 6:00 and 11:00 hr. On average, birds were bled 7.9 ± 4.3 min after capture (Range 2–15 min). Samples were stored on ice in the field until processing in the laboratory to separate plasma from cellular fractions. Plasma was separated and stored frozen at −20°C until immune assays were carried out.

For each bird, we assessed breeding status on the basis of brood patch occurrence (Redfern, 2010), and moult status on the basis of feather quality and occurrence of moulting primary wing feathers. Only females incubate eggs; therefore, only females could be classified as breeding or not. Since bulbuls are sexually monomorphic, we sexed all birds using gel electrophoresis. DNA extractions followed methods described by Richardson, Jury, Blaakmeer, Komdeur, and Burke (2001) and amplification was done using the P2/P8 primers (Griffiths, Daan, & Dijkstra, 1996).

### 2.2 Immune assays

#### 2.2.1 Haptoglobin concentration

We quantified plasma haptoglobin concentration using a functional colorimetric assay which quantifies the haem‐binding capacity of plasma. We followed instructions for the “manual method” provided with a commercially available assay kit (Cat. No.: TP801; Tridelta Development Ltd, Maynooth, Co. Kildare, Ireland). We used 7.5 μl of plasma and standard curves in respective wells. A five‐step serial dilution (2.5, 1.25, 0.625, 0.312, 0.156 and 0.08 mg/ml) of haptoglobin standard was used as the standard curve concentrations. We randomised samples before assays. We calculated within‐assay variability (*n* = 20 plates, maximum CV = 0.88, minimum CV = 0.31, mean CV = 0.49) and among‐assay variability (*n* = 807 samples, CV = 0.58) to verify consistency.

#### 2.2.2 Nitric oxide concentration

We measured nitric oxide concentration by a colorimetric assay described by Sild and Hõrak (2009). This estimates the concentration of nitrate and nitrite in plasma after reducing all nitrate to nitrite using copper‐coated cadmium granules. A measurable colour development proportionate to nitric oxide concentration follows reaction with Griess reagent. We used 10 μl of plasma for the nitrate reduction step. A five‐step serial dilution (100, 50, 25, 12.5, 6.25, 3.13 and 1.6 μM) of nitrate standard was used as the standard curve concentrations. We randomised samples before assays. We calculated within‐assay variability (*n* = 17 plates, maximum CV = 0.94, minimum CV = 0.48, mean CV = 0.70) and among‐assay variability (*n* = 667 samples, CV = 0.73) to verify consistency.

#### 2.2.3 Ovotransferrin concentration

Ovotransferrin was quantified by estimating the maximum amount of iron required to saturate all ovotransferrin in a sample. We used methods described by Horrocks, Irene Tieleman et al. (2011), following a three‐step process involving: saturation of ovotransferrin with ferric iron under alkaline conditions, reduction of excess unbound iron by ascorbic acid, then dissociation of ovotransferrin‐iron complex under acidic conditions, leading to colour development whose absorbance is measured by colorimetry. We used 10 μl of plasma and standard conalbumin concentrations (20, 16, 10, 4, 2 and 1 mg/ml) for test and standard curves, respectively. We randomised and analysed each sample in duplicate. We further calculated within‐assay variability (*n* = 48 plates, maximum CV = 1.18, minimum CV = 0.21, mean CV = 0.70) and among‐assay variability (*n* = 652 samples, CV = 0.53) to verify consistency.

All colorimetric assays (a–c above) were carried out using the Versamax plate reader (Molecular Devices, Sunnyvale, CA).

#### 2.2.4 Haemagglutination/haemolysis titres

We assessed natural antibody‐mediated haemagglutination and complement‐mediated haemolysis titres of plasma samples against 1% rabbit red blood cells (Envigo RMS (UK) Ltd.) in phosphate‐buffered saline as described by Matson et al. (2005). Twenty‐five microlitre of plasma was used as the starting concentration, and subsequent concentrations were obtained by a further 10‐step serial dilution of 25 μl plasma in 25 μl of phosphate‐buffered saline. 25 μl of 1% rabbit red blood cells was incubated in each well. Both haemagglutination and haemolysis titres were recorded as the number of serial dilution steps in which each function was still observable (i.e. 1:2 is 2 while 1:1,024 is 11; Matson et al., 2005). We randomised samples before assays. We calculated within‐assay variability (*n* = 255 plates, haemagglutination: maximum CV = 1.41, minimum CV = 0, mean CV = 0.43; haemolysis: maximum CV = 2.23, minimum CV = 0, mean CV = 1.48) and among‐assay variability (*n* = 801 samples, haemagglutination: CV = 0.51; haemolysis: CV = 2.38) to ensure consistency.

### 2.3 Statistics

We analysed each index separately because immune indices were uncorrelated or at most weakly correlated with each other (All *r* ^2^ < 5%), and a principal component analysis did not substantially reduce the number of variables (i.e. the five measured indices clustered into three principal components: PC1 (24%)—NO_x_ and haemolysis; PC2 (25%)—ovotransferrin and haemagglutination; and PC3 (21%)—haptoglobin, NOx, ovotransferrin and haemagglutination).

First, we tested the effects of environment and annual cycle stage on baseline innate immune function at the population level. We built general linear models for haptoglobin, NO_x_, ovotransferrin and haemagglutination, and a generalised linear model for haemolysis. We included season and annual cycle stage, and their interaction as predictor variables. We ran separate analyses for the two sexes because breeding status was only reliably determined for females. We grouped females into breeding (B), moulting (M) and neither (N) and excluded individuals that were breeding and moulting simultaneously (BM) because only three records were obtained for this group in the dry season (against 38 in the wet season). We grouped males into non‐moulting (NM) and moulting (M). For population level analyses, we used the first capture of an individual if it was caught more than once, to prevent pseudoreplication. Capture date—assigned as number of days from the first day of each season was included in each model to account for seasonal variation within the wet and dry seasons. 1 April was assigned the first day of the wet season while 1 November was assigned the first day of the dry season annually. Body mass was included in all models to account for the possible effect of individual body condition on immune indices. Haemolysis titre was modelled as a binary outcome throughout the study (0 or 1) because this was mostly zero or relatively low for most birds. Because samples were analysed in two batches (one for each year), we included “batch” as a fixed factor in all models. Significant effects of batch may result from natural differences between years and/or differences between the analyses (e.g. reagents, kits).

Secondly, we tested the effects of breeding and moult, and their interaction, on immune function within the wet season for females only. Again, we excluded repeat captures and included capture date and body mass in all models. We built general linear models for haptoglobin, NO_x_, ovotransferrin and haemagglutination, and a generalised linear model for haemolysis.

Finally, we tested whether the population level results occurred in the same way within individual birds, using individuals with multiple sampling records only. We built general linear mixed models for haptoglobin, NO_x_, ovotransferrin and haemagglutination, and a generalised linear mixed model for haemolysis, including individual identity as random factor in each model. We modelled within‐individual variation in each immune index using different data subsets, depending on availability, testing differences between seasons, breeding and moult states, keeping analyses separate for the sexes. To test differences between seasons, we only considered females that were neither breeding nor moulting, and males that were not moulting. To test differences between breeding and non‐breeding state and moulting and non‐moulting state, we considered individuals caught between these annual cycle stages within the wet season only. Further, we estimated individual repeatability using the package “rptr” (Stoffel, Nakagawa, & Schielzeth, 2017), including batch, season, breeding and moult as covariates in each model.

All models were reduced by a stepwise backward elimination, except for the repeatability models in which we retained season, annual cycle stage and batch even if they were not significant. However, we included the non‐significant outputs of all eliminated variables in our summary tables to show their performance in each model. We performed post hoc tests implemented by the package “lsmeans” (Lenth, 2016) to determine between group differences where interaction between predictor variables was significant. All statistical analyses were performed in r 3.4.4.

## 3 RESULTS

Overall, seasonal differences in immune function were more often explained by season than annual cycle stage (ACS) for all indices, except NOx concentration. The effect of annual cycle stages were largely only visible within the wet season. Aside some inconsistencies, results at the population level were largely similar to those within individuals that were sampled repeatedly.

### 3.1 Population level differences in immune function

#### 3.1.1 Between breeding and moult states in the wet and dry seasons

**Figure 1:**
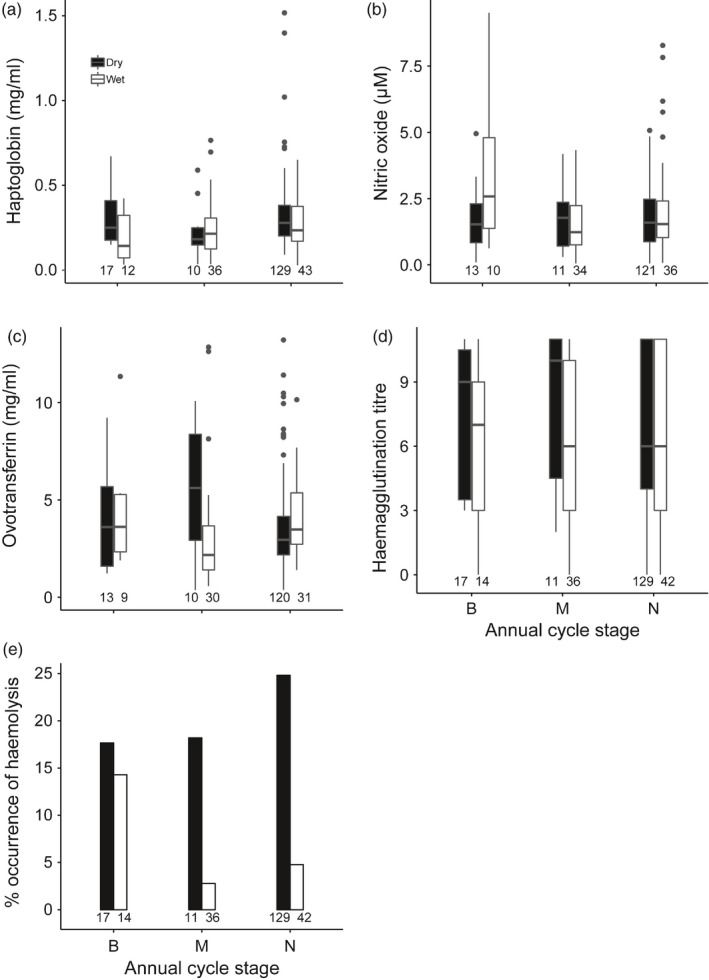
Seasonal differences in (a) haptoglobin, (b) nitric oxide, (c) ovotransferrin, (d) haemagglutination titre and (e) % occurrence of haemolysis between annual cycle stages in female Common Bulbuls, sampled over two annual cycles in Nigeria. Annual cycle stages were determined based on occurrence of brood patch and feather quality: B—breeding; M—moulting and N—non‐ breeding and non‐moulting. Sample sizes are indicated below each box/bar

In female bulbuls, ovotransferrin concentration, and haemolysis titre differed significantly between seasons (Figure 1). The effect of season on haptoglobin concentration was influenced by date (Table 1): haptoglobin concentration was always higher in the dry than the wet season (Figure 1a, post hoc test on dry season–wet season while controlling for date *t* = 4.0, *p* < 0.01), but increased with date through the dry season and remained low through the wet season (Supporting Information Figure S4). The haemagglutination titre did not differ significantly between seasons (Figure 1d, Table 1). Haptoglobin and ovotransferrin concentration and haemagglutination and haemolysis titre did not differ between annual cycle stages (Table 1). In contrast, variation in NO_x_ concentration was explained by an interaction between season and annual cycle stage (Table 1, Figure 1b)—NO_x_ concentration was only significantly higher for breeding females in the wet season (post hoc test: B–M (*t* = 3.2, *p* < 0.01), B–N (*t* = 2.0, *p* = 0.1), M–N (*t* = −2.1, *p* = 0.08)), but did not differ between annual cycle stages in the dry season (post hoc tests: all *p* > 0.94). Body mass did not explain variation in immune indices (Table 1).

**Table 1:** Population level differences in immune indices between annual cycle stages (ACS) between the wet and dry season in female and male Common Bulbuls *Pycnonotus barbatus*. Birds were sampled over two annual cycles in Nigeria. Sexes were analysed separately. Annual cycle stages were determined based on occurrence of brood patch and feather quality: breeding, moulting and non‐breeding and non‐moulting birds. Individuals sampled in both breeding and moult conditions were excluded from analyses. All individuals that were repeat sampled were also excluded

	Factor	*df*	HP		NO_x_		OVO		AGG		Lysis	
			*F*	*p*	*F*	*p*	*F*	*p*	*F*	*p*	LRT	*p*
Females	Batch	1	7.46	0.01	0.79	0.38	145.37	<0.01	4.59	0.03	1.15	0.28
	Body mass	1	3.06	0.08	0.02	0.89	0.55	0.46	0.43	0.51	1.02	0.31
	Date	1					1.24	0.27	2.68	0.10	2.33	0.13
	ACS	2	0.70	0.50		a	1.97	0.14	0.53	0.59	0.62	0.73
	Season	1		a		a	**6.78**	0.01**	1.77	0.18	**11.55**	<0.01***
	Season:ACS	2	0.47	0.62	**3.05**	0.04*	0.70	0.50	1.74	0.18	0.51	0.77
	Season:Date	1	**15.96**	<0.01***	**8.20**	<0.01*	0.15	0.70	0.00	0.97	0.21	0.64
Males	Batch	1	38.47	<0.01	0.03	0.86	82.80	<0.01	0.77	0.38	1.07	0.30
	Body mass	1	2.42	0.12	0.04	0.85	0.01	0.91	2.64	0.11	0.01	0.94
	Date	1					0.80	0.37	**16.92**	<0.01***	0.67	0.41
	Moult (M)	1	0.33	0.56	**4.41**	0.04*	0.02	0.89	0.26	0.61	1.10	0.29
	Season	1		a		a	0.13	0.71	**13.78**	<0.01***	**6.81**	0.01**
	M:Season	1	0.25	0.62	0.04	0.84	0.25	0.62	2.06	0.15	1.55	0.21
	Season:Date	1	**15.69**	<0.01***	7.19	0.01**	2.25	0.14	0.04	0.85	0.03	0.86

Notes

AGG: haemagglutination and lysis—haemolysis; HP: haptoglobin concentration; NO_x_: nitric oxide concentration; OVO: ovotransferrin concentration.

Summary statistics with significant *p*‐values are highlighted bold. * indicates level of significance: *p < 0.05; **p < 0.01; ***p < 0.001.

a Differences determined by post hoc tests due significant interaction between variables.

**Figure 2:**
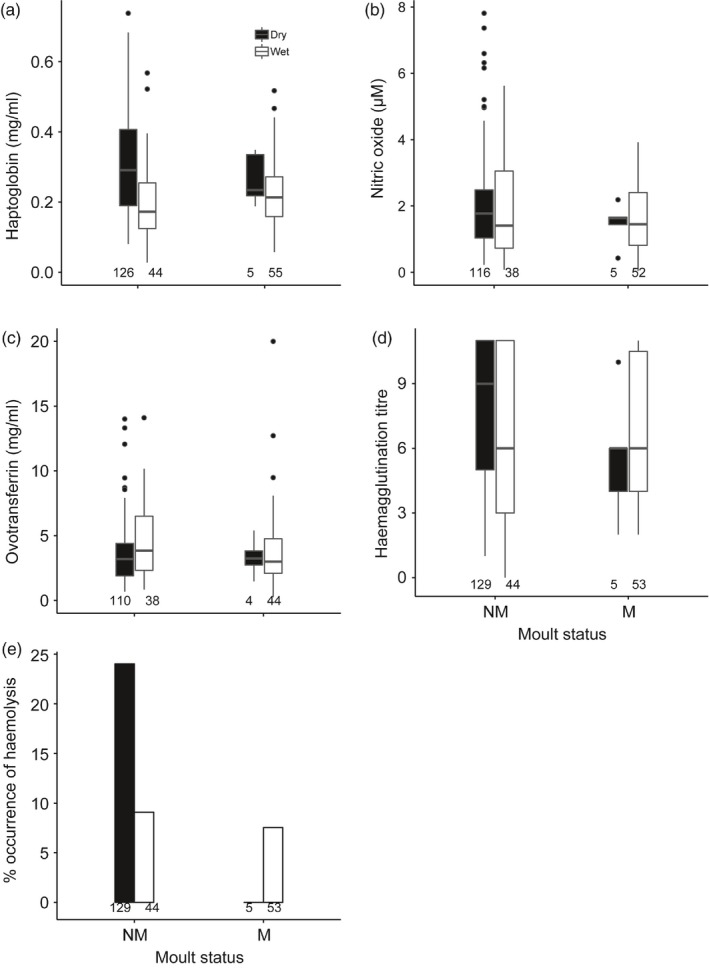
Seasonal differences in (a) haptoglobin, (b) nitric oxide, (c) ovotransferrin, (d) haemagglutination titre and (e) % occurrence of haemolysis between moulting and non‐moulting male Common Bulbuls sampled over two annual cycles in Nigeria. Annual cycle stages were determined based on feather quality: NM—non‐moulting and M—moulting. Sample sizes are indicated below each box/bar

Male bulbuls had significantly higher haemagglutination and haemolysis titres in the dry season, and like females, these indices did not differ between annual cycle stages (Figure 2, Table 1). Similar to females, the effect of season on haptoglobin concentration was influenced by date (Table 1): haptoglobin concentration was higher in the dry season than the wet season (Figure 2a, post hoc test on dry–wet season while controlling for date *t* = 4.0, *p* < 0.01), but increased significantly through the dry season and remained low throughout the wet season (Supporting Information Figure S5). Similarly, the effect of season on NO_x_ concentration was influenced by date: the difference between seasons was only significant after controlling for date (post hoc test: dry season – wet season *t* = 2.7, *p* < 0.01). NO_x_ increased during the dry season, but decreased during the wet season (Supporting Information Figure S5). Unlike in females, NO_x_ was significantly higher for non‐moulting compared to moulting males independent of season (Figure 2b, Table 1). Ovotransferrin concentration did not differ significantly between seasons and annual cycle stage in male bulbuls (Figure 2c, Table 1). Unlike females, haemagglutination increased significantly through the wet and dry season. Body mass did not explain variation in immune indices (Table 1).

#### 3.1.2 Between breeding and moult states in females within the wet season

**Figure 3:**
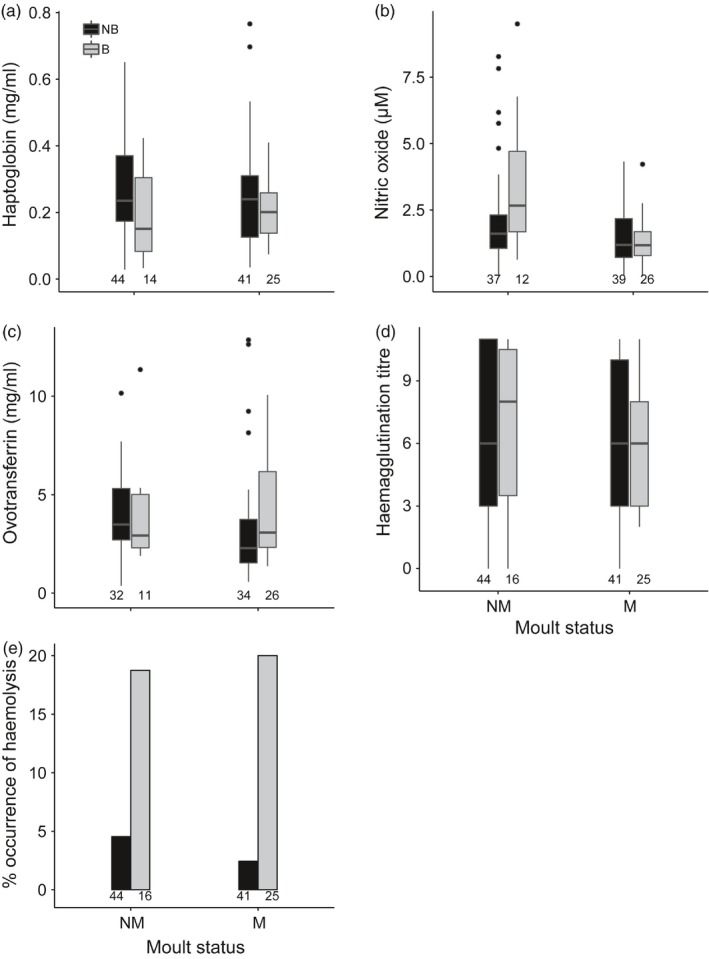
Differences in (a) haptoglobin, (b) nitric oxide, (c) ovotransferrin, (d) haemagglutination titre and (e) % occurrence of haemolysis between breeding and moult stages within the wet season in female Common Bulbuls sampled over two annual cycles in Nigeria. Annual cycle stages were determined based on occurrence of brood patch and feather quality: NM—non‐moulting; M—moulting, B—breeding and NB—non‐breeding. Sample sizes are indicated below each box/bar

Within the wet season, haptoglobin concentration (Figure 3a) was significantly lower, while ovotransferrin concentration (Figure 3c) and haemolysis titre (Figure 3e) were significantly higher in breeding compared to non‐breeding females. The moult state of female birds did not influence these immune indices (Table 2). The only exception was NO_x_ concentration (Figure 3b), where the interaction between breeding and moult was significant (Table 2). NO_x_ concentration was only significantly higher for breeding females in non‐moulting state (Figure 3b, post hoc (NM): NB – B (*t* = −2.5, *p* < 0.01))—during moult, breeding and non‐breeding females did not differ in NO_x_ concentration (Figure 3b, post hoc (M): NB – B (*t* = 0.1, *p* = 0.89)). Haemagglutination titre did not differ between breeding and non‐breeding females in the wet season (Figure 3d, Table 2). Body mass did not explain variation in immune indices (Table 1).

**Table 2:** Population level differences in immune indices between breeding and moult states in female Common Bulbuls *Pycnonotus barbatus* in the wet season. Bulbuls were sampled over two annual cycles in Nigeria. Breeding state was determined based on occurrence of brood patch and moult state based on feather quality. All individuals that were repeat sampled were also excluded

Factor	*df*	HP		NO_x_		OVO		AGG		Lysis	
		*F*	*p*	*F*	*p*	*F*	*p*	*F*	*p*	LRT	*p*
Batch	1	35.65	<0.01	2.17	0.14	39.63	<0.01	3.54	0.06	1.71	0.19
Body mass	1	0.27	0.60	0.20	0.65	0.01	0.92	0.00	1.00	3.21	0.07
Date	1	0.16	0.69	3.52	0.06	2.50	0.12	3.42	0.07	0.01	0.91
Breeding (B)	1	**15.07**	**<0.01** ***		a	**6.74**	**0.01** *	0.28	0.60	**7.36**	**<0.01** **
Moult (M)	1	1.25	0.26		a	0.03	0.86	0.98	0.33	0.48	0.49
B:M	1	0.35	0.56	**4.46**	**0.04** *	0.08	0.77	2.25	0.14	0.68	0.41

Note

AGG: haemagglutination and lysis—haemolysis; HP: haptoglobin concentration; NO_x_: nitric oxide concentration; OVO: ovotransferrin concentration.

Summary statistics with significant *p*‐values are highlighted bold. * indicates level of significance: ^*^ *p* < 0.05; ^**^ *p* < 0.01; ^***^ *p* < 0.001.

a Differences determined by post hoc tests due to significant interaction between variables.

### 3.2 Intra‐individual level differences in immune function

#### 3.2.1 Between seasons in non‐breeding and non‐moulting females, and non‐moulting males

**Figure 4:**
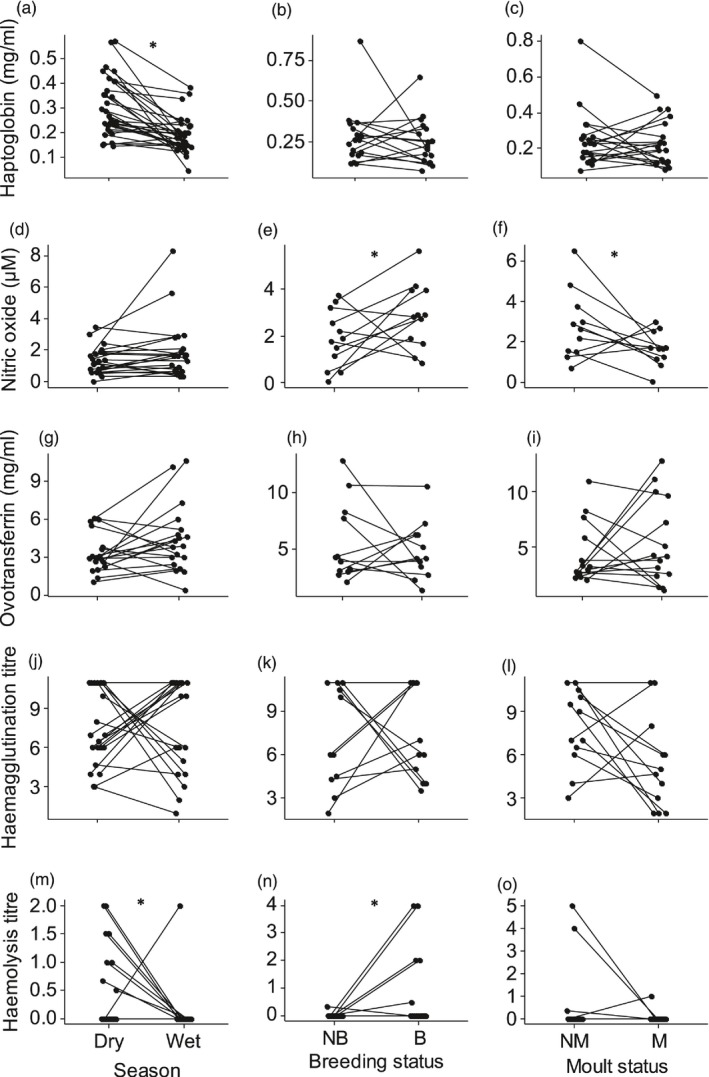
Within‐individual differences in (a–c) haptoglobin, (d–f) nitric oxide, (g–i) ovotransferrin, (j–l) haemagglutination titre and (m–o) haemolysis titre between wet and dry season, breeding and non‐breeding, and moulting and non‐moulting female Common Bulbuls. Breeding status was determined on the basis of brood patch occurrence: B—breeding and NB—non‐breeding. Moult status was determined based on feather quality: NM—non‐moulting and M—moulting. Immune indices with significant pairwise within‐individual difference are indicated by *

Within‐individual females, haptoglobin concentration and haemolysis titre were significantly higher in the dry season, while NOx concentration and haemagglutination titre did not differ between seasons (Figure 4, Table 3a). In contrast with the population level, ovotransferrin concentration did not differ between seasons (Figure 4g, Table 3a).

**Table 3:** (a) Summarised results of pairwise within‐individual comparison of immune indices in male and female Common Bulbuls: seasonal differences were compared for non‐moulting and non‐breeding females, and for non‐moulting males. Difference between breeding states was compared for females only in the wet season. Difference between moult states was compared for males and females in the wet season. Each factor was modelled separately using linear mixed models on each immune index. Factor: Group for which within‐individuals comparison was carried out. *N*: samples size used for analysis—number of individuals with repeat measures and total number of measures from all individuals. Chisq: test statistics from glmm. *p*: significance of test statistics (significant *p* values are highlighted bold). (b) Summarised results of individual repeatability in immune indices, estimated for each immune index and sex; controlling for season, breeding, moult state and batch effects. *N*: samples size used for analyses—number of individuals with repeat measures and total number of measures from all individual. Rpt: Individual repeatability estimate (significant repeatabilities are highlighted bold). CI: upper and lower confidence interval of repeatability estimates. Overall, difference between immune indices within individuals was consistent with differences at population level (Tables 1 and 2)

		HP			NO_x_			OVO			AGG			Lysis		
	(a)															
	Factor	*N*	Chisq	*p*	*N*	Chisq	*p*	*N*	Chisq	*p*	*N*	Chisq	*p*	*N*	LRT	*p*
Season	Females	31, 79	**7.06**	**0.01**	22, 54	1.02	0.31	19, 46	0.05	0.83	21, 56	0.08	0.78	28, 74	**3.87**	**0.04**
	Males	19, 48	**4.93**	**0.03**	13, 32	0.12	0.73	12, 30	0.08	0.77	16, 40	0.29	0.59	16, 40	0	0.97
Breeding	Females	17, 46	1.7	0.19	12, 32	**5.11**	**0.02**	12, 32	0.94	0.33	11, 30	0.04	0.83	16, 45	**7.63**	**0.01**
Moult	Females	20, 53	1.18	0.28	11, 31	**4.69**	**0.03**	15, 39	0.48	0.49	12, 34	3.16	0.08	19, 54	1.21	0.27
	Males	14, 36	0.03	0.87	9, 21	**6.25**	**0.01**	9, 21	0	0.98	5, 12	**5.67**	**0.02**	11, 26	1.24	0.26

	(b)															
		*N*	Rpt	CI	*N*	Rpt	CI	*N*	Rpt	CI	*N*	Rpt	CI	*N*	Rpt	CI
	Females	82, 216	**0.12**	0, 0.29	65, 161	**0.17**	0, 0.37	62, 151	0.07	0, 0.29	77, 182	0.03	0, 0.22	77, 204	0.02	0, 0.18
	Males	86, 216	0.01	0, 0.16	68, 164	**0.22**	0.04, 0.41	61, 147	0.07	0, 0.30	71, 183	**0.23**	0.04, 0.41	84, 209	0.01	0, 0.17

Note

AGG: haemagglutination and lysis—haemolysis; HP: haptoglobin concentration; NO_x_: nitric oxide concentration; OVO: ovotransferrin concentration.

Summary statistics with significant *p*‐values are highlighted bold.

**Figure 5:**
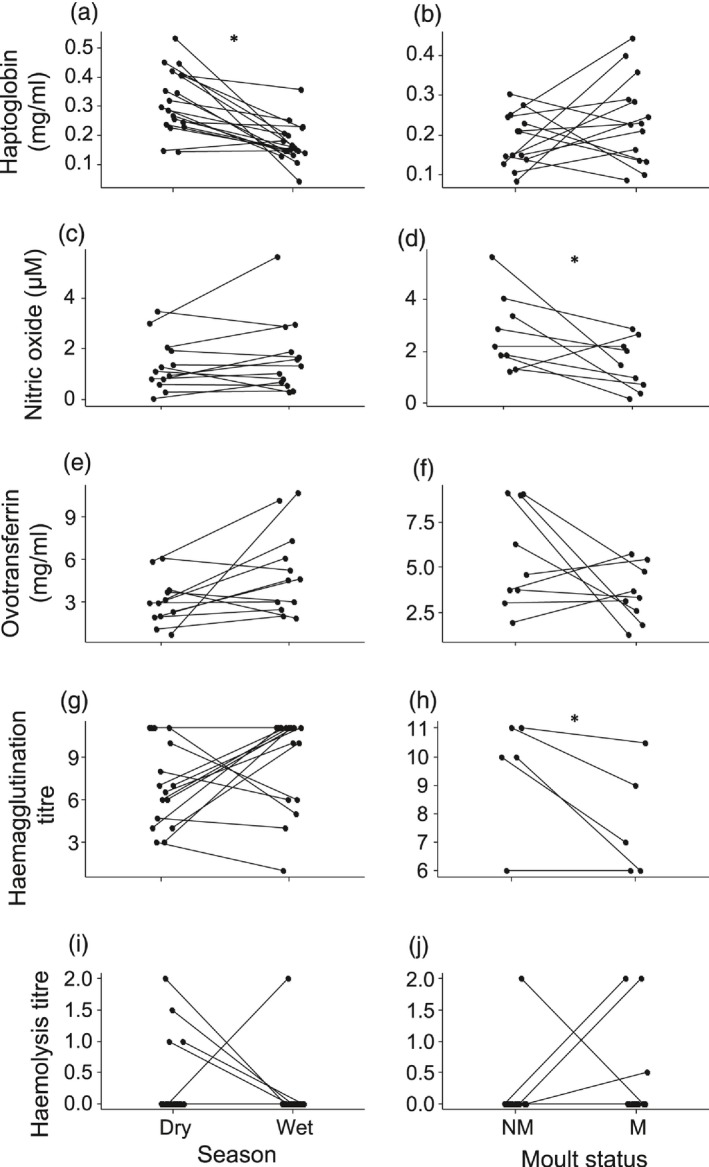
Within‐individual differences in (a, b) haptoglobin, (c, d) nitric oxide, (e, f) ovotransferrin, (g, h) haemagglutination titre and (i, j) haemolysis titre between wet and dry seasons, and moulting and non‐moulting in male Common Bulbuls. Moult status was determined on the basis of feather quality: NM—non‐moulting and M—moulting. Immune indices with significant pairwise within‐individual difference are indicated by *

Within‐individual males, haptoglobin concentration was significantly higher in the dry season, while NO_x_ and ovotransferrin concentration did not differ between seasons (Figure 5, Table 3a). Unlike at the population level, haemolysis titre did not differ between seasons, but individuals were more likely to have higher haemolysis titre in the dry season than in the wet season (Figure 5i, Table 3a).

#### 3.2.2 Between breeding and non‐breeding states in the wet season

Within‐individual females, NO_x_ concentration and haemolysis titre were significantly higher during breeding, while haemagglutination titre did not differ between breeding and non‐breeding states (Figure 4, Table 3a). Unlike at the population level, haptoglobin and ovotransferrin concentration did not differ significantly between breeding and non‐breeding states (Figure 4b,h, Table 3a).

#### 3.2.3 Between moulting and non‐moulting states in the wet season

Within‐individual females, NO_x_ concentration was significantly higher during moult, while haptoglobin and ovotransferrin concentrations, and haemagglutination and haemolysis titres did not differ between moulting and non‐moulting states (Figure 4, Table 3a).

Within‐individual males, NO_x_ concentration was significantly higher during moult, while haptoglobin and ovotransferrin concentrations, and haemolysis titres did not differ between moulting and non‐moulting states (Figure 5, Table 3a). Unlike at the population level, haemagglutination titre was significantly higher during moult (Figure 5h, Table 3a).

#### 3.2.4 Individual repeatability

Individual repeatability in immune indices was low to modest (0.01–0.23; Table 3b). Nonetheless, the repeatabilities for haptoglobin and NO_x_ concentrations were significant in females, while those of NO_x_ concentration and haemagglutination titre were significant in males (Table 3b).

## 4 DISCUSSION

In a system that decouples seasonal environmental variation and annual cycle stage in a tropical songbird, we found that seasonal differences in immune function were more often explained by environmental variation than annual cycle stage (Table 4). Immune indices were generally higher in the dry season than in the wet season, independent of annual cycle stage in both female and male birds. The only exception was NO_x_ concentration, which was higher for breeding females in the wet season only. We also found that within the wet season, breeding females were more likely to have higher immune indices than non‐breeding females, except for haptoglobin concentration which was significantly higher for non‐breeding females. Moult had no effect on immune function in the wet season. Patterns of immune variation at the population level were largely similar with patterns within individuals that were sampled repeatedly, except for a few inconsistencies, suggesting that population level variation reflects similar within‐individual variation and not simply among‐individual variation in immune function due to personality or quality (see results summarised in Table 4). Overall, our results suggest that innate immunity depends largely on the environment because, except for NO_x_, annual cycle stages were only significant within the wet season. Immune indices are often more variable within than among individuals as suggested by low repeatability, although some indices were still repeatable.

**Table 4:** Summarised population and individual level results showing cells with significant *p* values shaded grey

		Factor	HP	NOx	OVO	AGG	Lysis
			*p*	*p*	*p*	*p*	*p*
Population	Females	ACS	0.50		0.14	0.59	0.73
		Season			**<0.01**	0.18	**<0.01**
		Season:ACS	0.62	**0.04**	0.70	0.18	0.64
		Season:Date	**<0.01**	**<0.01**			
	Males	Moult	0.56	**0.03**	0.89	0.61	0.29
		Season		0.91	0.71	**<0.01**	**0.01**
		Moult: Season	0.83	0.86	0.62	0.85	0.21
		Season:Date	**<0.01**	**<0.01**			
	Females (WS)	Breeding	**<0.01**		**0.01**	0.6	**0.01**
		Moult	0.26		0.86	0.33	0.49
		Breeding: Moult	0.56	**0.04**	0.77	0.14	0.41
Individuals	Female	Breeding (WS)	0.19	**0.02**	0.33	0.83	**0.01**
		Moult (WS)	0.28	**0.03**	0.49	0.08	0.27
		Season	**0.01**	0.31	0.83	0.78	**0.04**
	Males	Moult (WS)	0.87	**0.01**	0.98	**0.02**	0.26
		Season	**0.03**	0.73	0.77	0.59	0.97

Note

AGG: haemagglutination and lysis—haemolysis; HP: haptoglobin concentration; NO_x_: nitric oxide concentration; OVO: ovotransferrin concentration; WS: indicates analyses within the wet season only.

Summary statistics with significant *p*‐values are highlighted bold.

Our finding of higher immune indices in the dry season independent of annual cycle stage is contrary to expectations based on predictions of lower infection risk with increased aridity (Horrocks et al., 2015; B. I. Tieleman, M. A. Versteegh, K. C. Klasing, & J. B. Williams, unpublished data) and lower investment in immunity under less optimal foraging conditions (French, DeNardo, & Moore, 2007). We found no indication that immune investment in Common Bulbuls was constrained by the occurrence of annual cycle stages—an explanation given for winter immune enhancement in temperate animals (Nelson & Demas, 1996). NO_x_ concentration was the only exception, and it is unlikely that its variation is due to competition for resources, because it was higher for breeding birds and lower for moulting birds. In seasonal environments like central Nigeria, one will predict that natural selection should shape immune function to counter predictable infection risks (Horrocks, Matson et al., 2011), and there are indications that vector‐ and waterborne diseases are typically high during tropical wet seasons (Altizer et al., 2006; Pascual et al., 2002; Young et al., 1993). But only few studies have investigated variation in immune function in tropical environments (Lee, Wikelski, Robinson, Robinson, & Klasing, 2008; Ndithia, Bakari, Matson, Muchai, & Tieleman, 2017; Tieleman et al., 2005), and none has done so over the entire annual cycle (Supporting Information Figure S2). Our study is also unique in that it takes place in a clearly seasonally arid environment, representative of the west African Savanna—a wide spread habitat where multiple factors may vary between and within seasons (Furley, 2006). Our results suggest that smaller scale local epidemiological factors such as seasonal habitat use, social interaction and diet shifts (Loehle, 1995; Sah, Mann, & Bansal, 2018) may have stronger impact on immune variation and possibly infection risk than factors related to general environmental productivity, which has been suggested as a proxy for antigen abundance (Horrocks et al., 2015). In support of this idea, incidences of free range poultry diseases in Africa are more prevalent in the dry season when environmental conditions are presumably harsh, causing birds to range more widely and cluster around fewer resources (Miguel et al., 2013; Nwanta, Egege, Alli‐Balogun, & Ezema, 2008). This may apply to many free living tropical animal species that cluster around resources in times of scarcity (Altizer et al., 2006), and to Common Bulbuls in particular, who we observe in large mixed species flocks around fruiting plants and water pools retained in gullies during the dry season (Brandt & Cresswell, 2008). Clearly, there is need to assess seasonal differences in infection status in addition to immune indices and to confirm whether enhancement of specific immune indices are in anticipation of, or response to, higher infection risk in the dry season.

Breeding and moult occurrence did not constrain investment in immune function as expected (Sheldon & Verhulst, 1996) or alternatively, enhanced immunity did not prevent breeding (Williams et al., 1999). However, the co‐occurrence of high haemolysis and NO_x_ concentrations coupled with lower haptoglobin concentrations in breeding compared with non‐breeding females during the wet season (Figure 3a,b,e) suggest that immune function may be organised differently for breeding and non‐breeding females. It could also mean that breeding females are more immunocompetent and/or less challenged than non‐breeding ones, or that challenged females omit breeding. The latter is likely the case if we interpret higher haemolysis titre and NO_x_ concentrations as enhanced capacity to destroy pathogens, and lower haptoglobin concentration to reflect lower infection. Alternatively, lower haptoglobin concentration in breeding birds may indicate a trade‐off between investing in breeding versus immune function (Råberg, Nilsson, Ilmonen, Stjernman, & Hasselquist, 2000). But lower haptoglobin concentration cannot be interpreted as an overall down‐regulation of immune function in breeding birds, because ovotransferrin and nitric oxide concentrations, and haemolysis titre were higher in breeding birds and haemagglutination titre was not affected by breeding state. Note however, that in a diet manipulation experiment on Common Bulbuls, we observed that low haemolysis titre and high haptoglobin concentration are associated with loss of body mass (C. J. Nwaogu, A. Galema, W. Cresswell, M. W. Dietz, & B. I. Tieleman, unpublished data), and breeding Common Bulbuls are more likely to be in better condition (Nwaogu et al., 2017), so high haemolysis titre and low haptoglobin concentration may indicate less challenge. Similarly, incubating Common Eiders *Somateria millissima* in better body condition have higher NO_x_ concentration (Bourgeon, Raclot, Le Maho, Ricquier, & Criscuolo, 2007). Perhaps, the immune system is re‐organised depending on annual cycle stage rather than out‐rightly down or up‐regulated (Buehler, Piersma, Matson, & Tieleman, 2008).

Our observation that moult or its overlap with breeding (Moreno, 2004; Sanz et al., 2004) did not explain variation in all measured immune indices (except for NO_x_ concentration) is largely consistent with the finding of Sandström, Prop, van der Jeugd, and Loonen (2014) where baseline immunity was associated more with date than moult stage in barnacle geese *Branta leucopsis*. But our results contrast those of Silverin, Fänge, Viebke, and Westin (1999) and Buehler et al. (2008) who suggested that breaking of feather follicles through the skin creates infected wounds that elevate immune function during moult. Unlike Sandström et al. (2014), we considered the entire moulting period a fixed annual cycle stage, comparable to breeding, and the absence of breeding and moult (see Figure 1). We do not expect different outcomes from using moult stage as a fixed or continuous variable, because in Common Bulbuls, moult correlates strongly with sampling date (Nwaogu et al., 2018), but except for haemagglutination in males, none of the measured immune indices correlated with sampling dates within the wet season when moulting largely takes place (Supporting Information Figure S4). Haemagglutination titre increased with sampling dates in males in both the wet and dry season, so the cause of variation in haemagglutination is not obvious. Overall, the occurrence of breeding and moult did not coincide with lower immune indices within the wet season. Nonetheless, we cannot conclude that there are no trade‐offs between immune function and the occurrence of breeding or moult. Rather, we propose that the effect of such trade‐offs may depend on environmental conditions (Ardia, 2005a), and this may not always lead to lower immune indices. Unfortunately, we could not statistically test the interactive effect of breeding and moult within the dry season because we sampled fewer birds breeding and moulting simultaneously. But plots of available data suggest that there are no differences in immune indices between annual cycle stages in the dry season (Supporting Information Figure S3).

Population and individual level patterns were largely consistent, and individual repeatabilities were low to modest. This similarity in trend between population and individual levels coupled with low individual repeatabilities for most immune indices implies that the impact of seasonal environmental variation and that of annual cycle stage within the wet season lead to larger variation within individuals than among individuals in the population (Lessells & Boag, 1987). There is no suggestion that population level patterns are resultant of among‐individual variation in immune function. However, significant individual repeatabilities for haptoglobin and NOx concentrations in females, and NO_x_ concentration and haemagglutination titre in male Common Bulbuls suggest that these indices are characteristic of individuals (Matson et al., 2012; Sild & Hõrak, 2009; Versteegh, Helm, Kleynhans, Gwinner, & Tieleman, 2014). If in addition to being repeatable, they are also heritable, as with other physiological traits like basal metabolic rate (Tieleman et al., 2009), they should be acted upon by natural selection (Falconer & Mackay, 1996), through the selective advantages they may offer individuals depending on environment.

Individual repeatability in some immune indices may account for some of the inconsistencies observed between population and individual level patterns, because population level analyses were carried out among individuals. Hence, if individuals are repeatable, there is a higher likelihood of finding population level differences even if such differences are not consistent within individuals, and the reverse may be the case if individuals are not repeatable. Nonetheless, some inconsistencies observed may still not be explained by individual repeatability. Patterns of variation in immune function are not always consistent across levels (Matson et al., 2006), and this may be the case between population and individuals. Moreover, immune indices were less variable within the wet season than the dry season (Supporting Information Figures S4 and S5), so wet season values may represent individual baseline. Future studies should therefore carefully select immune indices and sampling design depending on ecological question (Adamo, 2004).

Nitric oxide concentration, varying between annual cycle stages but not between seasons, deviated from the overall pattern observed for other immune indices. Elsewhere, H. K. Ndithia, M. A. Versteegh, M. Muchai, and B. I. Tieleman (unpublished data) also found higher NO_x_ concentrations in chick feeding red‐capped *Calandrella cinerea* and rufous‐naped larks *Mirafra africana* relative to non‐breeding ones in Kenya. Baseline NO_x_ concentrations in bulbuls (0–8 μM) were lower than baseline values in greenfinches *Carduelis chloris* (29–110 μM; Sild & Hõrak, 2009) and red‐capped larks (2–26 μM), but similar to those of rufous‐naped larks (2–6 μM; H. K. Ndithia, M. A. Versteegh, M. Muchai, & B. I. Tieleman unpublished data). NO_x_ concentration was also repeatable in bulbuls (Rpt = 0.17 (females) and 0.23 (males)) as observed for greenfinches (Rpt = 0.35; Sild & Hõrak, 2009). The reason why variation in NO_x_ concentration deviated from pattern observed for other immune indices in our study is not clear. NO_x_ is multifunctional, varying with physiological condition, health state, stress and work load (Bogdan, Röllinghoff, & Diefenbach, 2000; Bourgeon et al., 2007); hence, its concentration may be altered by other physiological processes related to breeding, moult or their interactions with the wet season.

## 5 CONCLUSIONS

By separating among from within‐individual variation in immune function in a natural study system where annual cycle stage is decoupled from seasonal environment, this study connects purely controlled laboratory experiments with population level field studies in ecological immunology (Pedersen & Babayan, 2011). We show that in a natural population seasonal environmental variation offers better explanation for seasonal differences in immune function than the occurrence of annual cycle stages or their associated costs, because breeding and moult were only significant in explaining immune variation under specific environmental conditions (i.e. within the wet season) and variation within individual was larger than among individuals for most indices. We provide evidence that variation in immune function within a single species in a single environment does not follow simple environmental productivity patterns, and this may also apply to disease risk. However, some inconsistencies between population and individual level patterns entail that caution is required when interpreting variation in immune function. A crucial next step is decomposing potential environmental effects into smaller measurable components such as diet, aridity, social contact, vectors and pathogen prevalence, using a combination of spatio‐temporal field observations and experiments. We recommend that explanations of seasonal variation in immunity should incorporate epidemiologically relevant ecological factors that reflect infection risk to individual animals under specific environments, and not be restricted to the costs of immune function (Hegemann, Matson, Versteegh, & Tieleman, 2012; Hegemann, Matson, Both et al., 2012) or the overall productivity of environments (Horrocks et al., 2015).

## AUTHORS’ CONTRIBUTIONS

C.J.N., B.I.T. and W.C. designed and raised funds for the project. C.J.N. was responsible for field data collection while B.I.T. and W.C. co‐supervised. C.J.N., B.I.T., M.A.V. and W.C. analysed data and interpreted results. C.J.N. developed the first draft of the manuscript. All authors read and approved the submission.

## Data Availability

Data for this article are deposited in Dryad Public Repository https://doi.org/10.5061/dryad.m559kb0 (Nwaogu, Cresswell, Versteegh, & Tieleman, 2018).
