# Supplementary material for: Seasonal differences in baseline innate immune function are better explained by environment than annual cycle stage in a year‐round breeding tropical songbird
Source: J Anim Ecol. 2019 Feb 6;88(4):537–53. doi: 10.1111/1365-2656.12948 (PMC6849850; doi:10.1111/1365-2656.12948)
Supplement: Supplementary file 1 [file JANE-88-537-s001.docx]

**Supplementary information**

**Seasonal differences in baseline innate immune function are better explained by environment than annual cycle stage in a year-round breeding tropical songbird.**

Chima J. Nwaogu^1, 2, 3^, Will Cresswell^2, 3^, Maaike A. Versteegh^1^ and B. Irene Tieleman^1^

^1^Groningen Institute for Evolutionary Life Sciences, University of Groningen, P.O. Box 11103, 9700 CC, Groningen, The Netherlands.

^2^ School of Biology, University of St Andrews, Harold Mitchell Building, St Andrews Fife KY16 9TH, UK.

^3^ A.P. Leventis Ornithological Research Institute, Jos, Nigeria.

*Correspondence: [c.j.nwaogu@rug.nl](mailto:c.j.nwaogu@rug.nl)

**Study species**

Common bulbuls are 25 – 50g sexually monomorphic resident passerines that can be found across Africa. They lay a clutch of typically two eggs. Their nesting period lasts between 30 to 34 days if successful, but fledglings remain in family groups supported by parents for up to 12 weeks post fledging (Nwaogu *pers obs*). Moult is largely seasonal and takes place in the wet season for most individuals in the population but some birds extend moulting into the dry season (Nwaogu et al. 2018). On average males commence moult 21 days earlier than females at the population level. Common bulbuls are largely frugivorous, but nestlings are predominantly fed insects and then fruits later. Adult birds also feed on insects, and occasionally on nectar or seeds. At the Amurum Forest Reserve in Nigeria, Common Bulbuls are territorial throughout the year but may move up to c. 2km to forage or drink from gullies in the dry season (Nwaogu *pers obs*).

**Study site**

The Amurum Forest Reserve (09°52’N, 08°58’E) is located at the A.P. Leventis Ornithological Research Institute on the Jos Plateau in north central Nigeria. It is a heterogeneous woodland savannah habitat with inselbergs and interspersed riparian forests, surrounded by farmlands and human settlements (Nwaogu and Cresswell 2015). It experiences a single wet and dry season annually. The wet season usually lasts from April to October (Fig. S1). Total monthly rainfall in the wet season is usually over 200 mm, but may be less in April, May and October, creating an annually predictable unimodal temporal pattern in humidity (Fig. S1). Minimum and maximum daily temperatures vary in a bimodal fashion due to increased cloud cover in the wet season and the movement of cold dry north-easterly trade winds from the Sahara to the Gulf of Guinea during the dry season between November and February. Overall, temperatures are lowest in the wet season between July and August and in the dry season between December and January. Daily temperature range is unimodal and lowest at the peak of the wet season around July and August (Fig. S1).

Environmental factors such as food, water and diet vary both between and within seasons. This is due to a combination of environmental heterogeneity, plant phenology and distribution, and the lengthy stretch of single wet and dry seasons. In the Amurum Forest Reserve few gullies in riparian forest fragments retain water in the dry season (Brandt and Cresswell 2008), and this usually serve as watering holes for bulbuls and other wildlife. Similarly, some plants flower and fruit during the dry season or in off peak periods of the year, attracting large numbers of insects and birds. Consequently, the number of animals around resources may vary according to the density, distribution or phenology of plants around the reserve, and may lead to a temporal shift in social contact (Nwaogu *pers obs*).

**References**

Brandt, M. J. and Cresswell, W. 2008. Breeding behaviour, home range and habitat selection in Rock Firefinches Lagonosticta sanguinodorsalis in the wet and dry season in central Nigeria. - Ibis 150: 495–507.

Nwaogu, C. and Cresswell, W. 2015. Body reserves in intra-African migrants. - J. Ornithol.: 1–11.

Nwaogu, C. J., Tieleman, B. I., & Cresswell, W. 2018. Weak breeding seasonality of a songbird in a seasonally arid tropical environment arises from individual flexibility and strongly seasonal moult. *Ibis*. https://doi.org/10.1111/ibi.12661

**Supplementary figures**

**Figure S1**: Environmental variability and occurrence of breeding and moult in the Common Bulbul over two annual cycles of wet and dry seasons from January 2014 to February 2016. Weather data was obtained from the Nigerian Metrological Agency at the Jos Airport located 26km from the A. P. Leventis Ornithological research Institute.

**Figure S2**: Spread of measured immune indices and body mass from 818 Common Bulbuls sampled over two annual cycles of wet and dry seasons from January 2014 to February 2016. All samples were collected from the A. P. Leventis Ornithological research Institute and analysed at the University of Groningen the Netherlands.

**Figure S3**: Differences in (A) haptoglobin, (B) nitric oxide, (C) ovotransferrin, (D) haemagglutination titre and (E) % occurrence of haemolysis between breeding and moult stages within the dry season in female Common Bulbuls sampled over two annual cycles in Nigeria. Annual cycle stages were determined based on occurrence of brood patch and feather quality: NM – non-moulting; M – moulting, B - breeding and NB - non-breeding. Sample sizes are indicated below each box/bar.


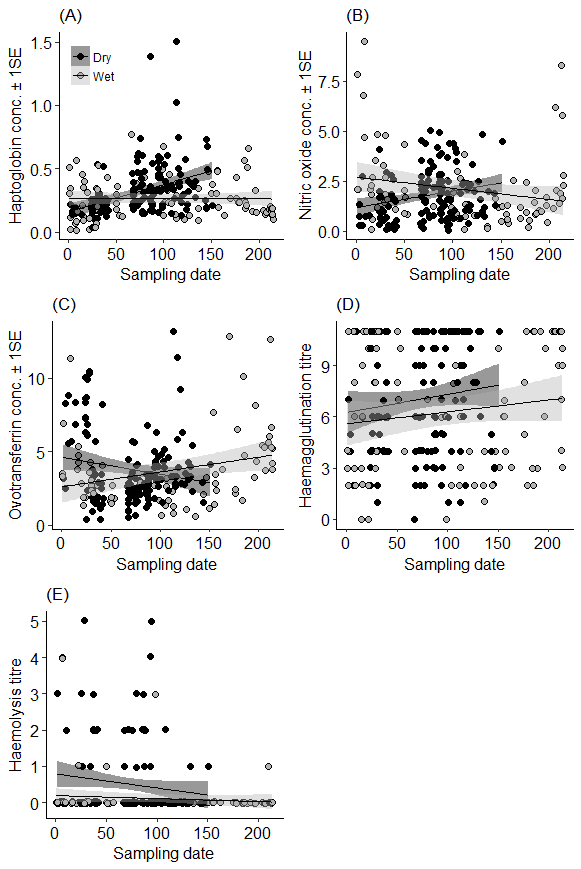


**Figure S4**: Variation in (A) haptoglobin, (B) nitric oxide, (C) ovotransferrin, (D) haemagglutination titre and (E) haemolysis titre within the wet and dry season in female Common Bulbuls sampled over two annual cycles in Nigeria. 1^st^ April was assigned the first day of the wet season while 1^st^ November was assigned the first day of the dry season annually.


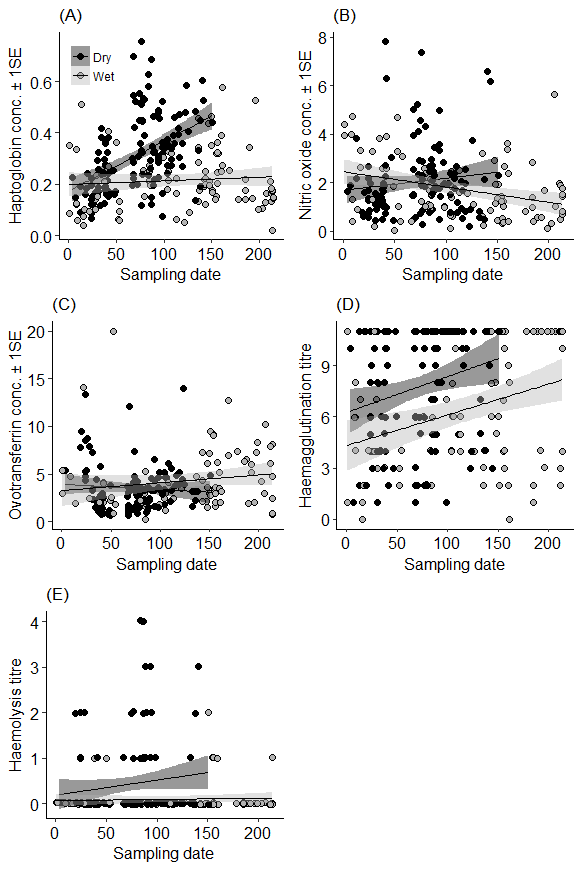


**Figure S5**: Variation in (A) haptoglobin, (B) nitric oxide, (C) ovotransferrin, (D) haemagglutination titre and (E) haemolysis titre within the wet and dry season in male Common Bulbuls sampled over two annual cycles in Nigeria. 1^st^ April was assigned the first day of the wet season while 1^st^ November was assigned the first day of the dry season annually.
